# Recurrent Postpartum Psychosis With Delayed Onset and Rapid Recovery: A Case Report

**DOI:** 10.7759/cureus.84506

**Published:** 2025-05-20

**Authors:** Gillian Jacobsen, Andrei Saffioti, Edmi Y Cortes, Vanessa L Padilla

**Affiliations:** 1 Medical Scientist Training Program, University of Miami Miller School of Medicine, Miami, USA; 2 Psychiatry and Behavioral Sciences, University of Miami Miller School of Medicine, Miami, USA

**Keywords:** bipolar disorders, mental health in pregnancy, postpartum mental health, postpartum psychosis, schizophrenia and other psychotic disorders

## Abstract

Postpartum psychosis is a serious mental illness characterized by psychotic symptoms, such as delusions or disorganized thinking, that develop shortly after giving birth. We present a case of a patient with no prior psychiatric history who experienced two episodes of postpartum psychosis, four months after giving birth for the first time and two months after giving birth for the second time. This case provides an unusual example of delayed onset and rapid recovery from postpartum psychosis. It also highlights the difficulty of determining the etiology of postpartum psychosis when a patient has no prior psychiatric history.

## Introduction

Postpartum psychiatric disturbances, such as “baby blues” or postpartum depression, are extremely common and thought to be mediated by biological, psychological, and social factors in the post-birth period. Postpartum psychosis (PPP) is perhaps the most severe psychiatric disturbance that may occur during this period. Unlike postpartum depression, which is typically characterized by persistent low mood, anhedonia, fatigue, or thoughts of self-harm or suicide, PPP is characterized by a complete break from reality, including confusion, paranoia, delusions, disorganized thought processes, and hallucinations, often mixed with mood symptoms [1]. Although PPP is not classified as a distinct diagnosis in the Diagnostic and Statistical Manual of Mental Disorders fifth edition text revision (DSM-5-TR), it is recognized as an episode of mood disorder with psychotic features or brief psychotic disorder using the specifier “with peripartum onset” for cases that occur during pregnancy or within four weeks postpartum [2].

Compared to “baby blues” or postpartum depression, the rate of PPP is relatively low, occurring in approximately one to two per 1,000 pregnancies [3,4]. Individuals diagnosed with bipolar I disorder or who have a history of PPP are at the greatest risk of experiencing PPP, with an overall postpartum relapse rate in these groups of 35% [5]. Other primary risk factors include first pregnancy, low socioeconomic status, family history of bipolar disorder or schizophrenia, sleep deprivation, traumatic birth experience, and lack of psychiatric treatment during pregnancy [6]. PPP is also associated with a high suicide and infanticide risk, and therefore, psychiatric hospitalization is typically warranted. We present a case of a patient who experienced two episodes of PPP occurring more than four weeks post-birth and largely resolving within days of treatment.

## Case presentation

A 23-year-old unemployed female with no psychiatric history presented to the mental health emergency department accompanied by her mother due to disorganized behaviors, bizarre delusions, and suicidal ideation. The patient presented four months postpartum after an uncomplicated vaginal delivery. The patient’s mother reported that starting two days after giving birth, the patient became depressed and withdrawn, not sleeping or eating, and relaying all childcare to her mother. Simultaneously, the patient was grieving the loss of her father, who had recently died, which the mother believed contributed to her symptoms. After three months of progressively worsening depressive symptoms, including suicidal ideation, the patient began displaying disorganized behaviors, hallucinations, and religious delusions. She believed “the Devil” was speaking to her, and she was found engaging in odd behaviors, trying to “purge the Devil.” She also exhibited potentially self-harming behaviors, such as removing knives from kitchen drawers and breaking glass objects. She did not communicate any infanticidal ideations, however, in fact stating that her desire to care for her baby was “the only thing keeping her from joining her father in heaven.” The patient was admitted to an inpatient psychiatric unit for further workup and treatment. 

The patient and her mother provided a full medical history, which included asthma and chronic anemia, but no psychiatric illness. Her only home medication was ferrous sulfate. She reported drinking alcohol daily “to help with sleep” but denied other substance use and was not exhibiting withdrawal signs. The patient’s mother reported a family history of depression in her mother and grandmother but denied any family history of suicide, psychosis, or bipolar disorder.

On admission, the patient was agitated, superficially cooperative, speaking incomprehensibly, and internally preoccupied (Table 1). She was initially treated with intramuscular dosages of haloperidol 5 mg, lorazepam 2 mg, and diphenhydramine 50 mg to target symptoms of psychosis, agitation, and aggression. The patient’s medical workup included a complete blood count, complete chemistry, thyroid-stimulating hormone level, vitamin B12 level, rapid plasma reagin, urine toxicology, and urinalysis. Substance-induced and medically induced psychosis were ruled out. The next day, the patient appeared withdrawn and guarded. She was started on escitalopram 10 mg daily to manage depressive symptoms. Within two days, the patient demonstrated rapid recovery. On day 3 of admission, she was found conversing with other patients with an improved affect. She described her mood as “pleasant” and denied any hallucinations or paranoid thoughts. On day 5, she continued to feel better and denied suicidal ideation. She reported sleeping and eating well and mentioned she just needed “a break from her routine life.” She was discharged with a 30-day prescription for escitalopram 10 mg daily. The patient did not follow up with outpatient psychiatric care.

**Table 1:** Summary of first admission

Timeframe	Signs and Symptoms	Treatment
Admission	Agitation, aggression, disorganized behavior, delusions, hallucinations, depressed mood, suicidal thoughts, self-harm	One-time dose of haloperidol 5 mg + lorazepam 2 mg + diphenhydramine 50 mg
Day 1-3	No psychotic symptoms, improving mood symptoms	Began escitalopram 10 mg daily
Day 4-5	No psychotic symptoms. Resolution of suicidal thoughts, improved mood and affect	Discharged with escitalopram 10 mg daily (30-day supply)

Two years later, the patient had another uncomplicated vaginal delivery followed by tubal ligation. She presented two months postpartum to the emergency department for symptoms of religious delusions and bizarre, disorganized behavior. According to the patient’s mother, the patient did not have depressive symptoms after this delivery but rather began having disorganized speech and behavior suddenly two days prior to presentation. After not showing up to work, the patient was found in her home “speaking in tongues” and smearing her menstrual blood on the walls as a way to “purge the Devil.” The mother reported that the patient did not continue psychiatric treatment after her prior admission and denied any new medical conditions or active substance use. The patient was admitted for psychiatric evaluation and treatment, with her mother serving as the healthcare proxy.

During this second admission, the patient was agitated and displayed disorganized behaviors of taking her clothes off and urinating on the floor. She again required intramuscular doses of haloperidol 5 mg, lorazepam 2 mg, and diphenhydramine 50 mg for agitation. Similar to her first admission, the patient’s urine toxicology was negative, and laboratory findings were unremarkable. During the first two days of admission, the patient was withdrawn, guarded, and paranoid. She was selectively mute and mostly slept, often refusing to eat or take medications. On day three, after three doses of risperidone 2 mg and valproic acid 500 mg, the patient displayed marked improvement. She was seen walking around the unit with improved mood, affect, and speech. She was cooperative during the interview and spoke with a normal rate and rhythm. She denied paranoia but maintained religious delusions and a lack of insight. She believed that she was in the hospital because she was “pushed from Heaven.” She also lacked insight into her first hospitalization, saying she was admitted previously because “a boy drugged [her].” On day four, due to concerns of medication adherence outside of the hospital, the patient received her first dose of long-acting injectable paliperidone palmitate (234 mg). By day five, her delusions were resolved, but her insight into her mental health and the reasons for both hospitalizations remained poor (Table 2).

**Table 2:** Summary of second admission

Timeframe	Signs and Symptoms	Treatment
Admission	Agitation, aggression, disorganized behavior, delusions, hallucinations, paranoia	One-time dose of haloperidol 5 mg + lorazepam 2 mg + diphenhydramine 50 mg
Day 1-2	Paranoia, selective mutism, guarding, withdrawal, depressed mood, delusions	Began risperidone 2 mg daily + valproic acid 500 mg daily
Day 3-5	Improved mood, resolution of paranoia, maintained delusions	One dose of paliperidone palmitate 234 mg Continued valproic acid 500 mg daily
Day 6-7	Resolution of psychotic and mood symptoms, but poor insight into illness	One dose of paliperidone palmitate 156 mg Discharged with valproic acid 500 mg BID (30-day supply)

Assessing the overall clinical presentation, the patient met diagnostic criteria for bipolar I disorder, the most recent episode being manic severe with psychosis. She was discharged on day seven after receiving the second dose of paliperidone palmitate (156 mg) via intramuscular injection and a prescription for valproic acid 500 mg orally twice a day. The patient and her mother were counseled on medication side effects and warning signs of illness recurrence. She was not breastfeeding prior to admission and had no desire to begin breastfeeding. She also did not require contraception as she had received a tubal ligation after her most recent delivery. The patient returned home upon discharge with full assistance from her mother.

## Discussion

Approximately 40% of patients experiencing PPP have no prior psychiatric history [1,4]. Thus, the underlying diagnosis that led to PPP often remains unclear. Studies indicate that at least 40% of patients who experience PPP have a one-time occurrence without any lasting psychiatric issues [6]. Among patients with a prior psychiatric history, PPP has the highest association with bipolar disorder [5,6]; therefore, early screening for bipolar disorder may help identify those most at risk of PPP. Underlying schizophrenia, schizoaffective disorder, or major depressive disorder with psychotic features are other possible diagnoses. Medical conditions and medication, or substance-induced psychosis, must also be ruled out in each case.

In our case, while the patient’s first presentation suggested major depressive disorder with psychotic features, her second presentation displayed no mood symptoms prior to the onset of psychosis, making the precise diagnosis difficult. The patient’s presentation of psychosis two to four months postpartum was also unusual, as 90% of PPP cases occur within two weeks of delivery [1]. Furthermore, the patient recovered quite rapidly with the initiation of psychotropic medications, considering the severity of her symptoms. The majority of her disorganized thoughts and behaviors resolved after emergency antipsychotic treatment during both admissions, while most episodes of PPP lasted weeks [1]. Overall, the patient met diagnostic criteria for bipolar I disorder, severe mania with psychosis, and responded well to the treatment that the patient consented to.

Treatment for PPP varies as it is typically based on the underlying psychiatric diagnosis. Possible treatment options include antipsychotics, benzodiazepines, mood stabilizers, and antidepressants [6,7]. The patient in this case responded to antidepressant treatment in her first episode, but due to a lack of depressive symptoms in her second episode, a combination of antipsychotic and mood stabilizing drugs was prescribed. One study suggests adding a mood stabilizer in addition to antipsychotic medication in the treatment of PPP due to the high association between PPP and bipolar disorder [3]. Valproic acid was chosen as a mood stabilizer for this patient because it was readily available in liquid form, which allowed for close monitoring of adherence; however, lithium would have also been appropriate, but the patient did not consent to lithium. Lithium has the best evidence of preventing suicide and infanticide related to mood disorders during the postpartum period [8]. Electroconvulsive therapy is another highly effective option, with an 87% remission rate when administered within six months of PPP onset [9].

In addition to proper medical treatment, it is crucial that patients treated for PPP have social support to help care for their newborn and to identify early warning signs of symptom recurrence, such as confusion or severe mood symptoms. This patient had strong psychosocial support, was living with her mother in stable housing, and was able to rely on family for childcare. Moreover, after the patient’s discharge from inpatient hospitalization for her second episode of PPP, outpatient follow-up was scheduled to monitor her symptoms and response to psychotropic medication. Psychiatric outpatient follow-up after initial hospitalization may minimize the risk of relapse. Altogether, while patients with PPP have better outcomes compared to patients with other causes of psychosis, outcomes are sequentially worse after more than one episode of PPP [10].

## Conclusions

This case report demonstrates that PPP can occur without any prior psychiatric diagnoses and may recur in this context. PPP may also present months after birth, outside of the typical four-week postpartum window when mood or psychotic symptoms tend to develop. It is important to screen for a history of mood symptoms or psychosis prior to delivery and closely follow postpartum patients to diagnose PPP early and prevent sequelae. By increasing awareness about PPP and offering targeted interventions, obstetricians and psychiatrists can significantly contribute to improving maternal mental health outcomes, bolstering mother-infant bonding, and supporting overall family well-being.
